# Mesenchymal Stromal Cell Differentiation for Generating Cartilage and Bone-Like Tissues In Vitro

**DOI:** 10.3390/cells10082165

**Published:** 2021-08-22

**Authors:** Graziana Monaco, Yann D. Ladner, Alicia J. El Haj, Nicholas R. Forsyth, Mauro Alini, Martin J. Stoddart

**Affiliations:** 1 AO Research Institute Davos, Regenerative Orthopaedics Program, 7270 Davos Platz, Switzerland; graziana.monaco@aofoundation.org (G.M.); yann.ladner@aofoundation.org (Y.D.L.); mauro.alini@aofoundation.org (M.A.); 2 Guy Hilton Research Centre, School of Pharmacy and Bioengineering, Keele University, Stoke-on-Trent, Staffordshire ST4 7QB, UK; n.r.forsyth@keele.ac.uk; 3 Institute for Biomechanics, ETH Zurich, Lengghalde 5, CH-8008 Zurich, Switzerland; 4 Healthcare Technology Institute, Institute of Translational Medicine, University of Birmingham, Birmingham B15 2TT, UK; A.ElHaj@Bham.ac.uk

**Keywords:** osteogenesis, chondrogenesis, donor comparison, osteochondral constructs

## Abstract

In the field of tissue engineering, progress has been made towards the development of new treatments for cartilage and bone defects. However, in vitro culture conditions for human bone marrow mesenchymal stromal cells (hBMSCs) have not yet been fully defined. To improve our understanding of cartilage and bone in vitro differentiation, we investigated the effect of culture conditions on hBMSC differentiation. We hypothesized that the use of two different culture media including specific growth factors, TGFβ1 or BMP2, as well as low (2% O_2_) or high (20% O_2_) oxygen tension, would improve the chondrogenic and osteogenic potential, respectively. Chondrogenic and osteogenic differentiation of hBMSCs isolated from multiple donors and expanded under the same conditions were directly compared. Chondrogenic groups showed a notable upregulation of chondrogenic markers compared with osteogenic groups. Greater sGAG production and deposition, and collagen type II and I accumulation occurred for chondrogenic groups. Chondrogenesis at 2% O_2_ significantly reduced ALP gene expression and reduced type I collagen deposition, producing a more stable and less hypertrophic chondrogenic phenotype. An O_2_ tension of 2% did not inhibit osteogenic differentiation at the protein level but reduced ALP and OC gene expression. An upregulation of ALP and OC occurred during osteogenesis in BMP2 containing media under 20% O_2_; BMP2 free osteogenic media downregulated ALP and also led to higher sGAG release. A higher mineralization was observed in the presence of BMP2 during osteogenesis. This study demonstrates how the modulation of O_2_ tension, combined with tissue-specific growth factors and media composition can be tailored in vitro to promote chondral or endochondral differentiation while using the same donor cell population.

## 1. Introduction

Cartilage and bone are unique and complex tissues, in physical proximity to each other but very different in structure, function, cell phenotype, microenvironment and mechanical stiffness. Bone and cartilage together form the osteochondral unit, which provides the structure and support necessary for load-bearing and movement. Damage to either of these tissues, caused by trauma or diseases such as osteoarthritis, reduces function, eventually leading to joint immobility and severe pain [1,2]. Depending on the nature, size and location of the osteochondral lesion, invasive surgical treatments are often required [3,4], including osteochondral grafts and mosaicplasty [5,6]. As an alternative, cell-based regenerative approaches such as autologous chondrocyte implantation (ACI) [7] or matrix assisted ACI [8] have been developed. However, the difficulties in first repairing the bone prior to applying ACI, joint orthofibrosis, and suboptimal osteochondral tissue structure, combined with the limited supply of donor tissue and, hence, low number of donor chondrocytes, can present significant clinical hurdles [9].

The limitations of autologous transplantation have heightened interest in the development of new tissue-engineering approaches for the treatment of cartilage and bone defects, separately, or as a combined osteochondral unit. Tissue-engineered approaches, that are mainly based on the use of cells with a scaffold carrier, aim to produce in vitro an autologous patient-tailored graft to restore osteochondral damage without the need for tissue harvesting [10,11]. The cell type used is a critical element that drives tissue repair or regeneration, both in vitro and in vivo after implantation. Several tissue-engineering strategies use primary chondrocytes, since this cell-type has the ability to create and maintain the desired tissue with positive outcomes [12,13]. However, to avoid sacrificing further cartilage tissue, other readily available sources need to be considered. One solution could be the use of primary autologous human bone marrow derived mesenchymal stromal cells (hBMSCs) [14]. hBMSCs can be easily harvested and isolated from bone marrow aspirates without the creation of a second cartilage injury, and since the cells belong to the same patient for whom the therapy is intended, there are no issues relating to rejection. Besides these advantages, hBMSCs are the best described, the most advanced in clinical use and, most importantly, have the ability to undergo both chondrogenic and osteogenic differentiation [15,16,17]. In addition, MSCs also overcome the problem of low donor cell number, as they maintain multilineage potential following expansion, allowing a sufficient cell number for engineered tissues to be achieved [14,18,19]. Thus, hBMSCs are an attractive cell source for osteochondral tissue engineering starting from a single batch of easily harvested and expanded autologous cells [20,21,22,23].

Different strategies have been attempted to engineer osteochondral tissue in vitro using MSCs. Usually, the osteochondral composite consists of a bone construct underlying a cartilage construct. In accordance with the classification proposed by Martin et al., osteochondral constructs have been generated using: (1) scaffolds for the bone zone with a scaffold-free approach for cartilage zone; or (2) the scaffolds for bone and cartilage are combined at the time of the implantation, or (3) a single heterogeneous composite, or (4) a single homogeneous scaffold for both zones [10].

The variety of models so far investigated highlight that despite significant advances in this area, a better understanding of the factors controlling MSC fate in the process of cartilage and bone formation will be crucial. Modulation of these factors could lead to a more stable and long-lasting tissue. This is particularly true when aiming for chondrogenic differentiation, where a key limitation exists due to MSC hypertrophy that can lead to bone formation instead of stable hyaline cartilage [24,25,26]. Appropriate environmental stimuli that can stabilize the articular chondrocyte phenotype have been studied and low oxygen tension ranging from 2% O_2_ to 5% O_2_ has been proposed to suppress cartilage hypertrophy [27,28,29]. This could relate to the similar oxygen tension found in vivo within articular cartilage, which ranges between 1% to 4%. However, cartilage and bone require different oxygen tensions and the development of an osteochondral composite by simultaneously exposing the cells to two different oxygen levels, requires a complex system that is not universally available [30]. Alternatively, the development of a single osteochondral unit requires the use of a unique culture media capable of successfully addressing MSC chondrogenic and osteogenic differentiation simultaneously. To the best of our knowledge, a unique culture media with these characteristics has not yet been developed

Therefore, we developed in parallel, separate in vitro constructs for bone and cartilage from the same passage of hBMSCs, with the approach to combine the two tissues at the time of the implantation. Due to donor variation, we believe it is crucial to study the various combinations on the same donor-derived cell source in parallel.

hMSCs seeded in fibrin:polyurethane scaffolds were treated with either chondrogenic culture media supplemented with TGFβ1 [31] or osteogenic differentiation media with or without bone morphogenic protein 2 (BMP2) [32]. Additionally, low and high oxygen conditions were compared.

We hypothesized that a cell-type-specific culture media, associated with the most suitable oxygen tension can be helpful in providing the right stimuli for more stable hBMSC chondrogenic and osteogenic differentiation from the same cell population. The overall aim of this study was to investigate the influence on culture conditions on chondrogenesis and osteogenesis from the same population of monolayer-expanded cells and characterize the different phenotypes that develop.

## 2. Materials and Methods

### 2.1. Poly(Ester-Urethane) Scaffold Preparation

Poly(ester-urethane) porous scaffolds (PU macroporosity ranging from 90 to 300 µm) were prepared using hexamethylene diisocyanate, poly (1-caprolactone) diol and isosorbide diol (1,4: 3,6-dianhydro-D-sorbitol) via a salt leaching-phase inverse technique [33]. The PU scaffold was cut by water-jet (CUTEC AG, Basel, Switzerland) producing cylindrical scaffolds (8 mm dimeter × 2 mm height) sterilized in a cold cycle at 37 °C via ethylene oxide process and degassed under vacuum for six days before use.

### 2.2. Isolation of Human Bone Marrow Derived MSCs

Bone marrow was obtained with full ethical approval (KEK-ZH-NR: 2010–0444/0) and the written consent from patients undergoing routine operations due to bone fracture. MSCs were isolated from four different marrow aspirates (one female 25-year old and three males 19, 50 and 66 years old) using Ficoll density separation (Sigma-Aldrich, Buchs, Switzerland).

Mononuclear cells were collected from the interphase seeded at a density of 50,000 cells/cm^2^ in alpha-minimum essential medium (αMEM) (Gibco, Carlsbad, CA, USA), 10% MSC tested Fetal Bovine serum (FBS) (Pan Biotech, Aidenbach, Germany), 5 ng/mL basic fibroblast growth factor (bFGF) (Peprotech, Rocky Hill, CN, USA) and 1% penicillin/streptomycin (Gibco). Media was refreshed after 96 h. The cells were passaged when 70% confluent and seeded at a cell density of 3000 cells/cm^2^. The chondrogenic potential of each donor was confirmed using standard techniques. hBMSCs isolated from each donor were used separately in four independent experiments.

### 2.3. Scaffold Seeding and Chondrogenic Differentiation

hMSCs at passage 3 were trypsinized, suspended in 75 μL of fibrinogen-thrombin-solution and seeded in cylindrical (8 mm × 2 mm) macroporous polyurethane (PU) scaffolds (fibrin provided by Baxter, Vienna, Austria) [34]. The number and distribution of the cells in each scaffold varied by group: (1) the scaffolds intended for osteogenic differentiation were evenly seeded with a cell density of 2 × 10^6^ cells/75 μL fibrinogen-thrombin-solution; (2) the scaffolds intended for chondrogenic differentiation were asymmetrically seeded in two steps: first, 3 × 10^6^ cells/75 μL fibrinogen-thrombin-solution were evenly seeded within the scaffold, then 500,000 hMSCs were resuspended in 100 µL of culture media and allowed to adhere to the upper surface of the scaffold in the incubator at 37 °C for one hour [34,35].

To achieve chondrogenic differentiation, constructs were kept in high glucose DMEM, 1% Insulin-Transferrin-Selenium (ITS), 1% Penicillin/Streptomycin, 1% non-essential amino acid, 50 μg/mL ascorbate-2-phosphate, 5 μM ε-amino-caproic acid (EACA), 10^−7^ M dexamethasone and 10 ng/mL TGF-β1 (Fitzgerald, Acton, MA, USA).

To achieve osteogenic differentiation, constructs were kept in low glucose DMEM supplemented with 1% Pen/Strep, 50 μg/mL ascorbate-2-phosphate, 5 μM ε-amino-caproic acid (EACA), 10 nM dexamethasone, 5 mM β-glycerol phosphate, 10% Fetal Bovine serum. In an additional group, 100 ng/mL Chinese hamster ovary (CHO) derived BMP2 was added to the osteogenic culture media.

**Figure 1:**
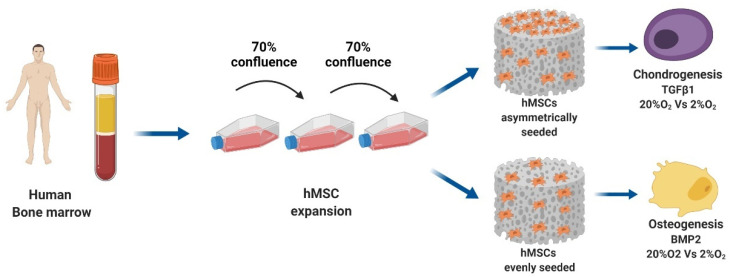
Schematic overview of the experimental design. Created with BioRender.com.

Constructs were exposed to either high (20% O_2_) or low oxygen (2% O_2_) using a small hypoxia chamber (BioSpherix, ProOx Model C21) that allowed for fast recovery of low oxygen tension every time the chamber was opened. The scaffold constructs were incubated for 14 days as described in the graphical abstract. The culture medium was changed every second day, and conditioned medium was collected three times per week for biochemical analysis. Prior to each media change, the media was conditioned to 2% O_2_ to minimize the O_2_ variation where required. A full experimental schematic is presented in Figure 1.

### 2.4. Gene Expression Analysis: RNA Isolation, cDNA Synthesis, Real Time qPCR

After 14 days of culture, constructs were harvested and total RNA was isolated using TRI Reagent (Molecular Research Centre Inc, Cincinnati, OH, USA). Total RNA was isolated at day 0 to assess basal gene expression levels.

TaqMan reverse transcription was then performed using 1 µg of total RNA, random hexamer primers and TaqMan reverse transcription reagents (Applied Biosystems, Carlsbad, CA, USA). Real-time PCR was performed using the QuantStudio 6 Flex real-time PCR system (Applied Biosystems). A panel of human genes associated with chondrogenic markers (COL2A1, ACAN, SOX9), the hypertrophic marker, COL10A1 and osteogenic markers (RUNX2; ALP; OC) were investigated.

Primers for RPLP0, COL2A1, COL10A1, ACAN, RUNX2, VEGF and OC mRNA were synthesized by Microsynth AG (Balgach, Switzerland) (Table 1). Primers for SOX9, SRY (sex determining region Y)-box 9 cartilage transcription factor (Hs00165814_m1), HIF1α (Hs00153153_m1) and ALP, alkaline phosphatase (Hs00758162_m1) were purchased as assays on demand from Applied Biosystems (Warrington, UK).

Relative quantification of target mRNA was determined according to the comparative CT method with hRPLP0 as endogenous control. In addition, the level of gene expression for each gene was determined relative to day 0 monolayer via a ΔΔCT comparison.

### 2.5. Sulphated Glycosaminoglycans and DNA Quantification

After 14 days of culture, constructs were digested with 1 mL proteinase K 0.5 mg/mL at 56 °C for 16 h. After digestion, total DNA content was measured spectrofluorometrically using Bisbenzimide Hoechst 33,258 dye (Polysciences Inc., Warrington, PA, USA) with purified calf-thymus DNA, as standard (Lubio Science, Luzern, Switzerland) [36].

Sulphated glycosaminoglycan (GAG) retained within the scaffolds was determined by the dimethylmethylene blue-dye method (Sigma-Aldrich, Buchs, Switzerland) at pH 3, using bovine chondroitin 4-sulfate sodium salt from bovine trachea (Fluka, St. Louis, MO, USA) [37]. The total GAG content of the culture media was also measured to estimate the release of matrix molecules from the constructs.

### 2.6. Histology and Immunohistochemistry

After 14 days of culture, 70% methanol-fixed constructs were frozen in OCT cryocompound Jung GmbH, Nussloch, Germany) before being sectioned (10 μm thick) on a cryotome (Carl Zeiss AG, Oberkochen, Germany).

### 2.7. Safranin O Staining

Safranin O staining was performed to detect proteoglycan rich areas. Sectioned samples were incubated for 12 min in Weigert’s Haematoxylin (Merck, Whitehouse Station, NJ, USA). The slides were then placed in lukewarm tap water for 10 min, then briefly washed in distilled water and placed in 0.02% (*v/v*) Fast Green (Fluka, St. Louis, MO, USA) in 0.01% (*v/v*) acetic acid in deionized water for 5 min. Fast green staining was followed by 30 s in 1% (*v/v*) acetic acid and then 5 min in 0.1% (*w/v*) Safranin O solution (Chroma-Gesellschaft Schmid GmbH & Co, Münster, Germany). The slides were then dehydrated in 96% ethanol 2 × 1 min and then 100% ethanol twice (2 min each). Slides were then placed in 100% xylene for 2 × 2 min before being mounted using Eukitt mounting medium.

### 2.8. Von Kossa Staining

Sectioned samples were stained with Von Kossa for 30 min in 5% (*v/v*) Silver Nitrate (Sigma Aldrich, Sigma-Aldrich, Buchs, Switzerland) with parallel exposure to strong light. The slides were then rinsed with deionized water and placed in 5% (*v/v*) Sodium Thiosulfate (Sigma Aldrich, Sigma-Aldrich, Buchs, Switzerland) for 10 min. The sections were washed again with deionized water and counterstained for 10 min with 0.1% (*v/v*) Nuclear fast red (Fluka, St. Louis, MA, USA). The slides were then dehydrated in 70% ethanol for 10 s, 96% ethanol for 1 min and then 100% ethanol twice (2 min each). Slides were placed in 100% xylene for 2 × 2 min before being mounted using Eukitt mounting medium.

### 2.9. Immunostaining Collagen Type I and II

The presence of collagen types I and II was determined using the following primary antibodies: COL-1 (C2456; Sigma-Aldrich) and CIICI (Developmental Studies Hybridoma Bank, University of Iowa, Iowa City, IA, USA).

Sections were brought to room temperature and washed in distilled water for 10 min before native peroxidase activity was blocked with 0.3% H_2_O_2_ (Fluka) in methanol (Brenntag) for 30 min. Sections were then air-dried and washed twice for 5 min in 0.1% Tween-20 PBS.

Sections were incubated in 0.05–0.5 units/mL hyaluronidase (Sigma-Aldrich) in 0.1% Tween 20 PBS at 37 °C for 30 min before being washed three times for 5 min in 0.1% Tween-20 PBS. Sections were then blocked with horse serum diluted 1:20 in 0.1% Tween-20 PBS for 1 h at room temperature. Following the blocking step, the serum was removed without washing and immediately replaced with the primary antibody for an incubation time of 30 min at room temperature.

The following dilutions with 0.1% Tween-20 were used for the antibodies: COL1 1:2000 and CIICI 1:6. Negative controls were incubated with 0.1% Tween20 PBS. Slides were then washed three times for 5 min with 0.1% Tween-20 PBS, before being incubated with the biotinylated anti-mouse IgG secondary antibody (Vector Laboratories, Burlingame, CA, USA) diluted 1:200 in 0.1% Tween-20 PBS. Following this incubation, sections were washed again in 0.1% Tween-20 and then incubated with ABC solution (Vector Laboratories) for 30 min at room temperature, washed again, and then incubated with ImmPACT DAB (Vector Laboratories, Burlingame) for 4 min before being placed into distilled water. Samples were then counterstained with Mayer’s haematoxylin (Sigma-Aldrich) for 20 s and blued in tap water for 5 min. Samples were dehydrated in 50%, 70%, 96%, 100%, 100% ethanol before being cleared in xylene and mounted with Eukitt (Sigma-Aldrich).

### 2.10. Statistical Analysis

The data were produced from four individual experiments, each carried out with hMSCs from a different donor. All experiments were performed in triplicate and quadruplicate for each group at different timepoints in order to reduce methodological variability. Each measurement was performed in duplicate. Analyses were conducted between the appropriate control group and treatment groups, as well as between different treatment groups. The Shapiro–Wilk normality test was used to determine if data sets exhibited a normal distribution before statistical analysis. Where data were normally distributed, one-way ANOVA with Tukey’s post hoc testing was applied. For data sets that did not show a normal distribution, the Friedman test with the uncorrected Dunn’s test was applied.

A significance level of *p* < 0.05 was applied and data are presented as Mean and Standard Deviation. Analyses were carried out using the GraphPadPrism 8.1 software (GraphPad Software Inc., La Jolla, CA, USA)

## 3. Results

Due to donor variation, studying osteogenesis and chondrogenesis in parallel, with the same population of cells, can aid in the understanding of specific factors supplemented in the media and their interaction with oxygen tension. With this in mind, we analyzed the gene expression of the same cell population after 2 weeks of different stimuli.

### 3.1. Gene Expression Analysis

**Figure 2:**
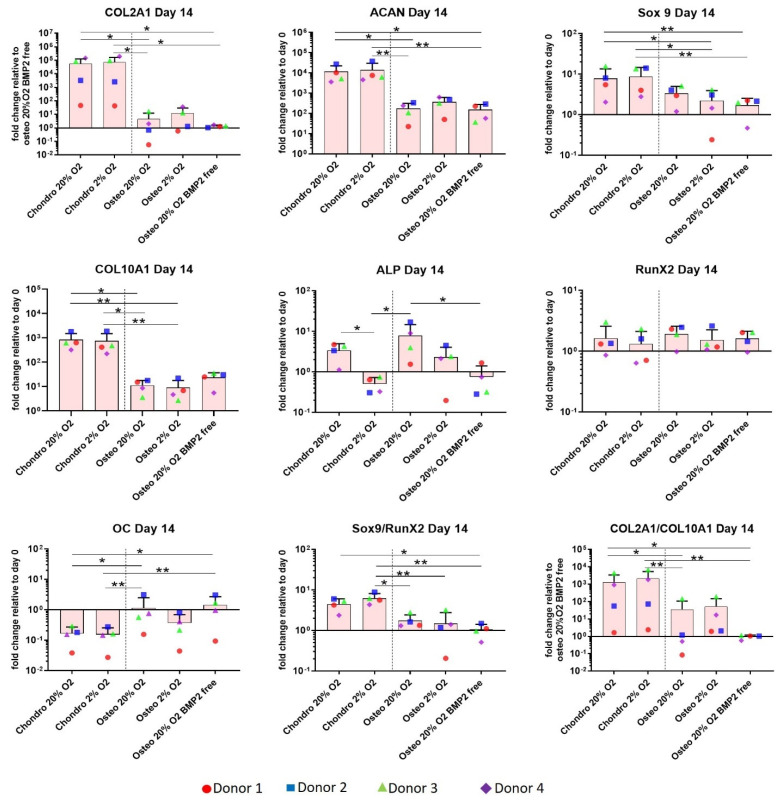
Gene expression measured at day 14 by real-time polymerase chain reaction (qPCR) of chondrogenic and osteogenic scaffolds. A significant upregulation of the chondrogenic markers Collagen type II, Aggrecan and SOX9, and the hypertrophic marker Collagen type X, was observed in the chondrogenically differentiated constructs compared with osteogenic samples. The Sox9/Runx2 and Col2A1/Coll10A1 ratios were also greater in chondrogenic samples. Furthermore, ALP gene expression was consistently downregulated in chondrogenically differentiated constructs under 2% oxygen tension. An upregulation of ALP was observed in the osteogenically differentiated constructs, with the exception of BMP2 combined with 20% O_2_. Statistical significance was defined as * *p* < 0.05 and ** *p* < 0.01.

A panel of genes associated with chondrogenic differentiation (Collagen type II, Aggrecan, SOX9), hypertrophy (Collagen type X and alkaline phosphatase (ALP)), and osteogenic differentiation (RUNX2, Osteocalcin (OC)) were investigated on day 14 (Figure 2). The four donors which were investigated all displayed the same pattern of expression to a varying degree of magnitude.

Relative quantification of target mRNA was performed according to the comparative Ct method. COL2A1 is relative to BMP2 free osteogenic condition as it was not expressed by all donors on Day 0. Values represent the mean and standard deviation of four independent hBMSC donors in experimental triplicate. Statistical significance was defined as * *p* < 0.05 and ** *p* < 0.01.

Collagen type II and aggrecan show a very similar gene expression profile and a significant upregulation of these two genes was observed in the chondrogenic groups, particularly between (1) chondro 20% O_2_ and osteo 20% O_2_ (* *p* < 0.05), and (2) chondro 2% O_2_ and osteo 20% O_2_ (* *p* < 0.05 or ** *p* < 0.01), independently of the presence or absence of BMP2 (Figure 2). SOX9 showed a similar profile to collagen type II and aggrecan, with the exception that significant differences in the osteo-chondro group comparison were detected between the chondro groups and Osteo 2% O_2_ (* *p* < 0.05). Osteogenic differentiation led to lower SOX9 expression than chondrogenic groups independent of oxygen tension. Osteo 20% O_2_ BMP2 free showed a significantly lower SOX9 expression than the chondrogenic groups (** *p* < 0.01).

The hypertrophic marker collagen type X showed a significant upregulation in the chondrogenically differentiated groups compared with the osteogenically differentiated groups, and differences were independent of the oxygen tension applied (* *p* < 0.05 and ** *p* < 0.01). However, the absence of BMP2 from the osteogenic media led to a loss of significance in the osteo-chondro comparison due to a slight increase in collagen X expression in the osteo 20% O_2_ BMP2 free group. ALP expression was downregulated under 2% O_2_ compared to 20% O_2_ for both chondrogenesis and osteogenesis in the presence of BMP2, although this only reached significance for chondrogenesis (* *p* < 0.05).

In three out of four donors, RUNX2 was downregulated during chondrogenesis under low O_2_, although no statistically significant differences were detected as donor 2 actually had an increase in RUNX2 expression. The gene expression ratios SOX9/and Collagen type II/Collagen type X were significantly upregulated RUNX2 (* *p* < 0.05 or ** *p* < 0.01) in the chondrogenic groups. The differences between chondro and osteo groups were more marked when chondro groups were differentiated under lower O_2_ (** *p* < 0.01) than 20% O_2_ (* *p* < 0.05). These last two results indicate that low O_2_ is beneficial, leading to more reproducible increases in the SOX9/RUNX2 and Collagen type II/Collagen type X ratios.

**Figure 3:**
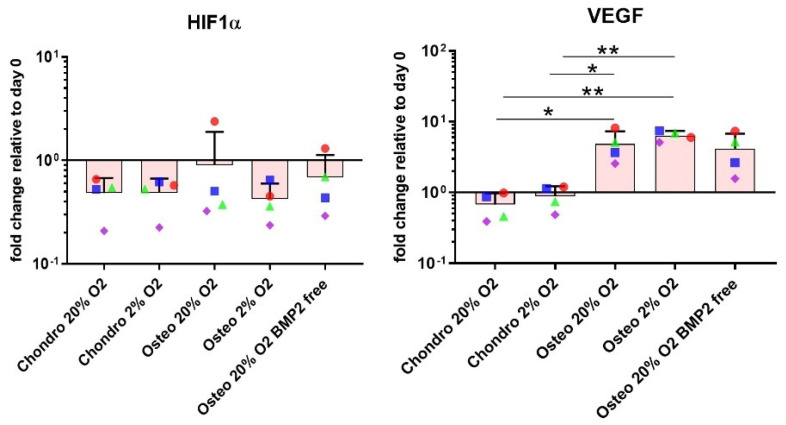
Gene expression measured at day 14 of differentiation by real-time polymerase chain reaction (qPCR). VEGF gene expression was significantly upregulated in the osteogenic groups supplemented with BMP2. Relative quantification of target mRNA was performed according to the comparative Ct method. Values represent the mean and standard deviation of four independent hBMSC donors in experimental triplicate. Statistical significance was defined as * *p* < 0.05 and ** *p* < 0.01.

The data shows that while chondrogenic differentiation behaved generally as expected, osteogenic medium in the absence of BMP2 combined with 3D culture conditions showed an expression pattern that may be more indicative of endochondral ossification, as indicated by the higher SOX9/RUNX2 and Collagen type II/Collagen type X ratios. Osteocalcin was significantly upregulated in the osteogenic groups under 20% O_2_ independently of the addition of BMP2 when compared with the chondrogenic groups (* *p* < 0.05 and ** *p* < 0.01). Conversely, the 2% O_2_ osteogenic group tended to suppress OC gene expression (Figure 2). VEGF expression was unaffected by oxygen tension but was significantly upregulated under osteogenic conditions (Figure 3). HIF1α was largely unaffected by all conditions investigated. (Figure 3).

### 3.2. Sulphated Glycosaminoglycan and DNA Quantification

**Figure 4:**
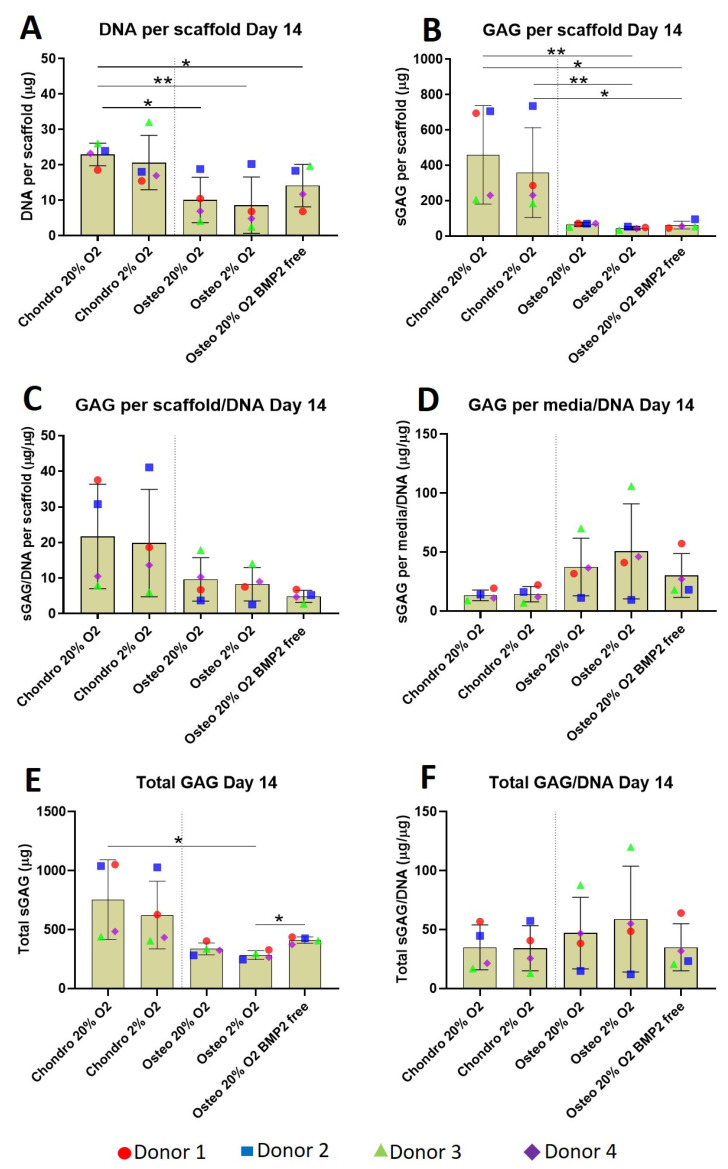
Biochemical analysis of chondrogenically and osteogenically differentiated constructs after 2 weeks in culture shows a significantly higher sulphated glycosaminoglycan deposition in chondrogenically differentiated constructs independent of oxygen tension. (**A**) Bisbenzimide Höchst 33528 dye was used to quantify the DNA in proteinase K digests of scaffolds. (**B**–**F**) Dimethylmethylene blue (DMMB) at pH 3 was used to determine the sulphated glycosaminoglycan (GAG) produced by mesenchymal stem cells (MSCs): (**B**) sulphated glycosaminoglycan produced by MSCs and deposited inside the scaffold: (**C**) GAG/DNA per scaffold: (**D**) culture media GAG normalized to DNA: (**E**) cumulative GAG deposited into the scaffold and released in culture media; (**F**) Total GAG/DNA. Values represent the mean ± SD of four independent hBMSC donors in experimental triplicate or quadruplicate. Statistical significance was defined as * *p* < 0.05, and ** *p* < 0.01.

As expected, due to the different seeding concentrations (3.5 versus 2 × 10^6^ cells/scaffold), after 2 weeks in culture, the DNA content of chondro 20% O_2_ was significantly higher compared to all osteogenic groups (Figure 4A). Although the DNA content of the chondro 20% O_2_ group is similar to 2% O_2_, the latter did not reach significance due to the higher standard deviation. Absence of BMP2 in the osteogenic culture media slightly increased the DNA content of the osteo 20% O_2_ group but this increase is not statistically different to the other osteogenic groups.

After 14 days of differentiation, chondrogenically differentiated constructs consistently accumulated more sGAG inside the scaffolds compared with osteogenically differentiated constructs (Figure 4B). No oxygen tension or BMP2-related differences were observed. The scaffold GAG/DNA ratio (Figure 4C) was very similar in the overall profile to the sGAG per scaffold with a higher value for the chondrogenic groups compared with the osteogenic groups. Unexpectedly, the media GAG/DNA ratio (Figure 4D) showed an opposite trend, with the osteogenic groups showing a higher GAG content compared to the chondrogenic groups.

The amount of total GAG produced by the chondrogenic groups showed a higher average compared with the osteogenic groups (Figure 4E). However significant differences were identified only between the chondro 20% O_2_ and osteo 2% O_2_. The GAG level of the latter group was also significantly lower compared to the osteo 20% O_2_ BMP2 free group. Total GAG/DNA (Figure 4F) is very similar among the various groups with a slightly higher average value for the osteogenic groups supplemented with BMP2; the main difference was the location of the GAG, with chondrogenic retaining a greater proportion in the scaffold, and osteogenic retaining a greater proportion in the media.

**Figure 5:**
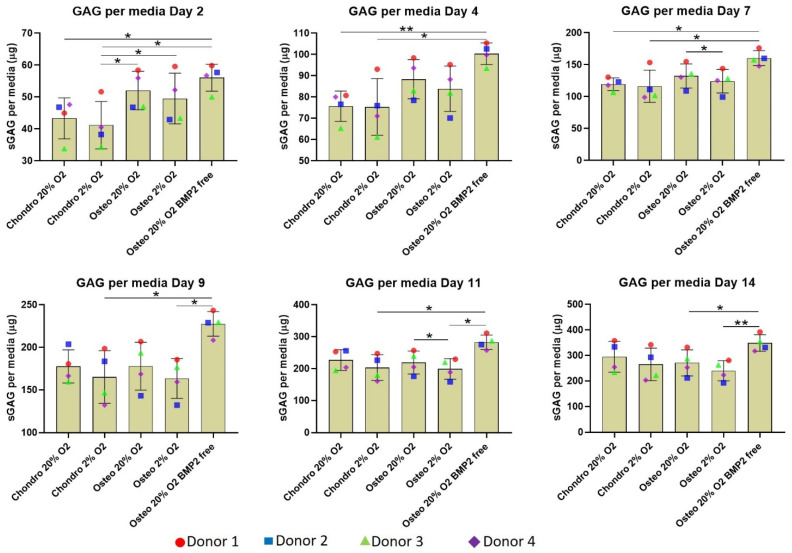
GAG produced and released into culture media from scaffolds over 2 weeks in culture. In the first 7 days, the osteogenically differentiated groups released significantly more GAG into the culture media compared with the chondrogenically differentiated constructs. The absence of BMP2 in the osteogenic media kept the media GAG higher until day 14. sGAG content in culture medium was determined spectrophotometrically following reaction with 1.9-dimethylmethylene blue (DMMB) pH 3. Values represent the mean ± SD of three independent hBMSC donors in experimental quadruplicate or quintuplicate. Statistical significance was defined as * *p* < 0.05 and ** *p* < 0.01.

A more detailed analysis of GAG release into the media over time showed the osteogenically differentiated groups released significantly more GAG into the culture media compared to the chondrogenically differentiated constructs during the first 7 days of differentiation (Figure 5). The difference is attenuated over time, although the osteo 20% O_2_ group lacking BMP2 continued to release a significantly higher amount of GAG into the media supernatant in the second week of differentiation. Thus, the chondrogenic media supplemented with TGFβ1 significantly facilitated differentiation, while the absence of BMP2 in the osteogenic culture media increased GAG release from day 4 onward.

### 3.3. Histology **&** Immunohistochemistry

**Figure 6:**
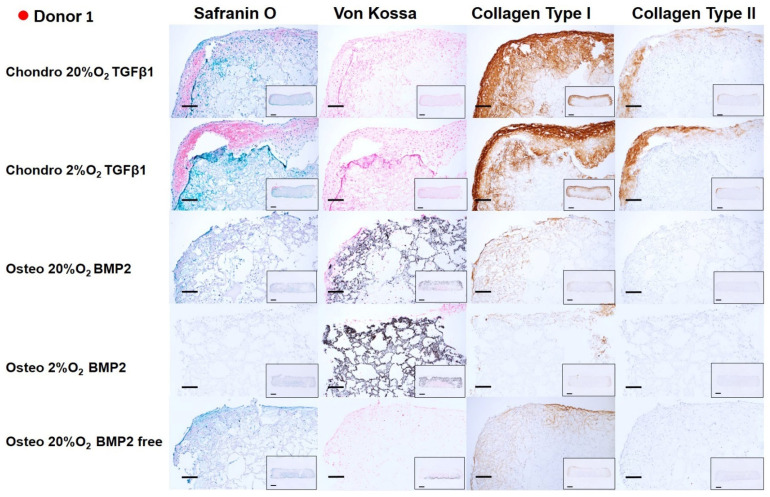
Chondrogenic and osteogenic hBMSC-constructs after 2 weeks of culture. Images showing a higher magnification of donor 1. Scale bar: 200 µm. Inset: Images showing the full section of the constructs 1. Scale bar: 1 mm. Scaffolds were stained with safranin O/Fast Green, Von Kossa and immunohistochemically labelled for collagen type I and collagen type II. Chondrogenically differentiated groups showed higher Safranin O, Collagen Type I and Collagen type II deposition. The osteogenically differentiated groups with BMP2 are positive for Von Kossa staining.

**Figure 7:**
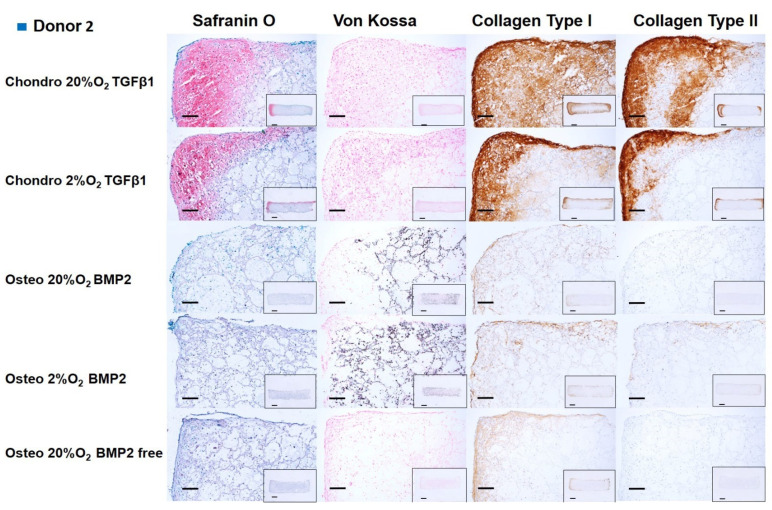
Chondrogenic and osteogenic hBMSC-constructs after 2 weeks of culture. Images showing a higher magnification of donor 2. Scale bar: 200 µm. Inset: Images showing the full section of the constructs 1. Scale bar: 1 mm. Scaffolds were stained with safranin O/Fast Green, Von Kossa and immunohistochemically labelled for collagen type I and collagen type II. Chondrogenically differentiated groups showed higher Safranin O, Collagen Type I and Collagen type II deposition. The osteogenically differentiated groups with BMP2 are positive for Von Kossa staining.

**Figure 8:**
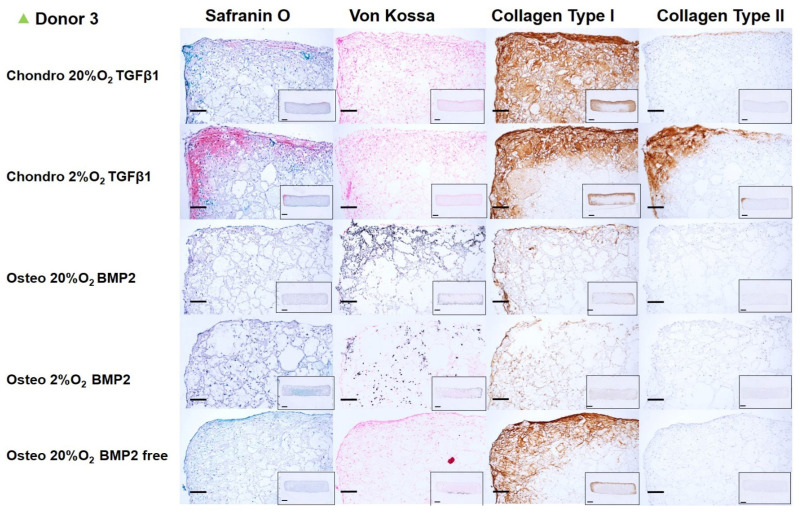
Chondrogenic and osteogenic hBMSC-constructs after 2 weeks of culture. Images showing a higher magnification of donor 3. Scale bar: 200 µm. Inset: Images showing the full section of the constructs 1. Scale bar: 1 mm. Scaffolds were stained with safranin O/Fast Green, Von Kossa and immunohistochemically labelled for collagen type I and collagen type II. Chondrogenically differentiated groups showed higher Safranin O, Collagen Type I and Collagen type II deposition. The osteogenically differentiated groups with BMP2 are positive for Von Kossa staining.

**Figure 9:**
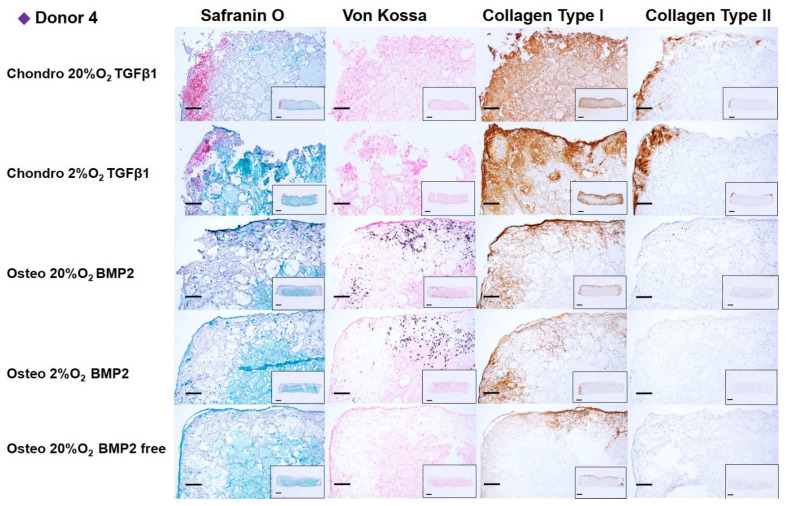
Chondrogenic and osteogenic hBMSC-constructs after 2 weeks of culture. Images showing a higher magnification of donor 4. Scale bar: 200 µm. Inset: Images showing the full section of the constructs 1. Scale bar: 1 mm. Scaffolds were stained with safranin O/Fast Green, Von Kossa and immunohistochemically labelled for collagen type I and collagen type II. Chondrogenically differentiated groups showed higher Safranin O, Collagen Type I and Collagen type II deposition. The osteogenically differentiated groups with BMP2 are positive for Von Kossa staining.

After 14 days of chondrogenic or osteogenic differentiation, samples were stained with Safranin O and counterstained with Fast green in order to detect sulphated GAGs deposition (Figure 6, Figure 7, Figure 8 and Figure 9). Positive Safranin O staining was present along the upper and lateral surface of the constructs differentiated with chondrogenic media, with the exception of Donor2 hypoxia tended towards on increased staining under low oxygen conditions. No Safranin O staining was observed in the osteogenically differentiated constructs under any condition.

Von Kossa staining highlighted a mineral deposition in the osteogenically differentiated constructs only when the media was supplemented with BMP2. None or very low mineral deposition was observed in the absence of BMP2 demonstrating, also in our system, that BMP2 enhances the osteogenic differentiation capacity of mesenchymal stromal cells derived from human bone marrow [38]. No relevant differences in mineral deposition were observed at the different oxygen tensions.

Collagen type I deposition was evident in chondrogenically differentiated groups, with reduced the collagen type I deposition under low oxygen. Osteogenically differentiated constructs showed an overall notable reduction in the collagen type I deposition compared with chondrogenically differentiated groups. The BMP2 supplemented osteogenic media did not affect the Collagen type I deposition. However, for donor 3, the absence of BMP2, led to an increased collagen type I deposition. Immunohistochemistry showed that collagen type II was found only in chondrogenically differentiated groups, similarly to safranin O staining, collagen II staining was increased under low oxygen, with the exception of Donor2.

## 4. Discussion

MSCs have been extensively explored as a source of cells, generating major clinical interest, and are easy to isolate without creating any morbidity site. They represent an emerging advanced-therapy medicinal product (ATMP) treatment [39,40]. Numerous studies have investigated the role of growth factors and oxygen tension on the differentiation of hMSCs. In this study, we provide a unique comparison of multiple conditions on multiple human donors. In the present work, autologous MSCs from four different human bone marrow donors were isolated and expanded in 2D culture following identical protocols. This approach provided a uniformly treated batch of MSCs that offered an initial common and unequivocal baseline from which to start the chondrogenic and osteogenic differentiation procedures. Therefore, after 14 days of 3D culture, we can directly compare the behavior and response of the same cell population towards the two differentiation protocols in separate constructs. When considering the data, it is noteworthy that osteogenic media is routinely used in 2D conditions such as monolayer. Differences observed in our system during osteogenesis may also be due to 3D-encapsulated cells being subjected to media typically used under 2D conditions. Thus, the same media may have different outcomes depending on the culture system used, and this should be further investigated in future studies.

As expected, our findings show that culture media and oxygen tension influences hMSC differentiation. Particularly at day 14, we consistently observed for the chondrogenically differentiated groups:

- A notable upregulation of chondrogenic gene expression markers and SOX9/RUNX2 and COL2A1/COL10A1 ratios.
- A consistent and significant downregulation of hypertrophic marker alkaline phosphatase (ALP) gene expression under low oxygen tension.
- A notably higher sGAG production and deposition within the constructs.
- Positive staining for safranin O, Collagen type I and II.
- Increased staining for safranin O and Collagen type II under 2% O_2_ in 3 from 4 donors.
- Slight reduction of the Collagen type I deposition under 2% O_2_.
- For the osteogenically differentiated groups:
- An overall upregulation of osteogenic markers gene expression.
- A significant upregulation of ALP in BMP2 supplemented media under 20% O_2_ tension.
- A slightly reduced upregulation of ALP and OC under 2% O_2_ tension.
- A very low sGAG deposition within the constructs.
- A significant increase in media sGAG in the absence of BMP2 at day 14
- 2% O_2_ reduced the ALP and OC gene expression.
- Positive Von Kossa staining only in the presence of BMP2, low collagen type I deposition and absence of collagen type II deposition in all groups.

### 4.1. Low Oxygen Tension Induces A Significant Downregulation of Alp Gene Expression in Chondrogenic Media

Several studies have shown that differences in oxygen tension can affect MSC differentiation with varying results [28,41]. High in vitro O_2_ tension negatively affects MSC chondrogenesis and results in the expression of the hypertrophic markers collagen Type X and ALP, which upon in vivo implantation in nude mice results in ectopic bone formation [42]. The chondrogenic fate of young porcine MSCs can be metabolically programmed by low oxygen tension to acquire an articular chondrocyte-like phenotype via mechanisms that resemble natural development [28]. Yet, studies with human cells have shown considerably more variation [28,41]. Furthermore, the response to oxygen has been shown to be material dependent. Human MSCs demonstrated reduced expression of ALP and collagen X at 1% O_2_ when encapsulated in low concentration Hyaluronan (HA) gels; yet, the opposite was true when the HA concentration was increased to 5% [43], with the authors speculating that the differences were due to the ability of the gel to retain pericellular matrix. At 5% O_2_, hBMSCs in alginate beads, which retain most of the de novo matrix, continued to show an increased expression of the chondrogenic marker SOX9, ACAN and Collagen type II and a decrease in the ALP and RUNX2, with donor dependent changes in Collagen type X [44].

Within our system, fibrin is not very efficient at retaining *de novo* matrix and our results were intermediate, with a consistent downregulation of ALP. The ALP data would be strengthened by additional protein data, but this was not performed. Of note, ALP has multiple functions, not all of which are bone related [45]. This opens the possibility that the observed changes are related to changes in metabolism, but more detailed analysis would be needed to investigate this aspect. In contrast with our ALP finding, we did not observe a downregulation of collagen type X expression under 2% O_2_ chondrogenesis. Although several studies have shown a reduction in collagen type X expression under low oxygen [46,47], other studies report a negligible effect or even an upregulation of collagen type X gene expression [48,49]. In this regard, our findings belong to the latter group of studies where a negligible effect has been observed. It would be interesting to confirm collagen type X deposition at the protein level in our experiments, however, this was not possible due to the lack of a suitable antibody that does not cross-react with the fibrin glue used in our constructs.

Despite the samples being contained within a validated hypoxic chamber, there was a lack of a difference in VEGF and HIF1α expression with low O_2_. The reasons for this are unclear, but it suggests a more robust O_2_ related response could be obtained. It has also been previously demonstrated that regulation of HIF1α in hMSCs under low oxygen is largely dependent on the underlying chondrogenic potential of the cells [46]. The lack of response suggests that the ALP gene expression changes seen in both chondrogenic and osteogenic conditions are not HIF1α driven. A further reason why the material used, and the implant size, may affect responses to oxygen tension could be due to the differing diffusion rates into the scaffold. Larger, or more dense scaffolds, with a high cell density may already be largely hypoxic due to diffusion limits and this may have played a role with our 8 mm × 4 mm scaffolds containing more the 2 million cells.

Osteocalcin was not markedly expressed at day 14, potentially because it is a later osteogenic marker. RUNX2 was not particularly affected by the differentiation process in either differentiation processes, although chondrogenic differentiation under low oxygen appeared to downregulate its expression for two donors. During osteogenesis, the downregulation of SOX9, rather than the upregulation of RUNX2, has been shown to be a more predictive marker of osteogenic potential when using human bone marrow derived MSCs. Under chondrogenic culture conditions the SOX9/RUNX2, an indicator of chondrogenic *versus* osteogenic differentiation, and COL2A1/COL10A1, an indicator of resting chondrogenesis *versus* endochondral ossification, ratios were significantly higher if compared with the osteogenic groups_._

### 4.2. Chondrogenic TGFβ1-Supplemented Media Promotes sGAG Deposition while Osteogenic Media without BMP2 Induces sGAG Release in the Culture Supernatant

Previous studies also reported an increased GAG and matrix production in chondrogenic cultures under low oxygen tension for pellet and 3D scaffold constructs using chondrocytes, articular cartilage progenitors and MSCs [43,50,51,52]. However, other studies did not identify significant increases in GAG production or reported a reduced matrix production under 1% to 5% O_2_ in hMSCs in pellet or scaffold cultures [53,54]. The different oxygen densities used in the present work did not exert any noticeable effect on GAG deposition, which is in line with the latter studies mentioned above.

In this study, the level of sGAG deposition inside the 3D MSC-based constructs was significantly higher in the chondrogenic constructs, presumably due to TGFβ1 supplementation [31,55,56]. In addition, sGAG content was also quantified in the culture media. We observed a significantly increased sGAG release into the culture media for all osteogenically differentiated groups from day 2. The release of sGAG was attenuated over time in BMP2 containing media. However, in the absence of BMP2, the high sGAG release was maintained until day 14. We are not aware of any reports investigating GAG production during osteogenesis in vitro. As the GAG production during 3D osteogenesis does appear to be significant, we propose this marker to be adopted for future 3D osteogenesis studies. In vivo, GAG production is observed during endochondral ossification. The release into the medium observed here may provide an indication that indirect versus direct differentiation is occurring. Of note, donor 2 and donor 3 showed an opposite behavior depending on which medium they are exposed to, thus highlighting the inherent problems of donor variation, with methods to predict cell function being needed [57]. Alternatively, a higher number of donors with non-statistical based methods of analysis will be needed. By studying multiple conditions with the same starting cell population of cells, as undertaken here, the donor variation can be better assessed, leading to more clinically relevant results.

### 4.3. TGFβ1- Supplemented Chondrogenic Media under 2% O_2_ Tension Promotes Cartilage-Like Matrix Deposition and Reduces Collagen Type I, While BMP2-Supplemented Osteogenic Media Promotes Matrix Mineralization

Chondrogenic differentiation of hMSCs led to positive staining by Safranin O, especially at the borders of the constructs and in the areas where a higher cell density was seeded following the asymmetric seeding approach previously described [35]. In addition to the asymmetric seeding, the higher matrix deposition at the edges of the constructs may have been influenced by the facilitated oxygen and nutrient exchange at the upper and side of the constructs rather than in the middle, or at the bottom edge that was in contact with the multi-well plate surface. However, metabolically active cells were present in the center of the construct, as shown by the mineralized matrix found throughout the whole scaffold, especially for donors 1 and 2, under osteogenic conditions. In the chondrogenic groups, collagen II deposition was identified in the same areas of the constructs positively stained by Safranin O, and both increased under 2% O_2_ in 3 from four donors. Similarly, to previous studies [46], the effect of low oxygen was dependent on the chondrogenic potential of the donor, with the most chondrogenic donor (Donor2) being the least responsive to low O_2_. Collagen type I deposition was detected in more extended regions of the scaffold’s edges. The low oxygen density (2% O_2_) reduced the collagen type I deposition compared to higher oxygen density (20% O_2_). Indeed, collagen type I staining was present on all edges and penetrated the scaffold to a larger extent under 20% O_2_. Although several studies suggest an increase in collagen type II gene expression or protein deposition under low oxygen condition, they also report a parallel increase in collagen type I. Other studies report a downregulation of matrix gene expression (SOX9, COL2A1, ACAN) with no effect on matrix formation [53,58,59].

Following 14 days of MSC osteogenic differentiation, mineralized matrix was positively stained by Von Kossa and a notable reduction in collagen I deposition was observed in comparison with the chondrogenic groups. In addition, a more marked reduction in collagen I was present in the same areas of the constructs where a higher degree of mineralization occurred. This inverse correlation between collagen I deposition and degree of mineralization is consistent in all the donors investigated. As expected, most of the mineralization occurred when BMP2 was supplemented in the culture media.

Although the ideal oxygen concentration for MSC chondrogenic differentiation has been broadly accepted as low, conflicting outcomes exist for defining the ideal oxygen tension to achieve a proper MSCs osteogenic differentiation. Osteogenic differentiation in a culture media supplemented with 100 ng/mL BMP2 with a very similar composition to that used in this study, appeared to be inhibited under 1% O_2_ while the increase to 3% O_2_ restored osteogenesis [60]. In contrast to previous studies, Wagegg et al., showed hypoxia as a promoter of hBMSC osteogenesis, suggesting a positive effect of low oxygen found in fracture hematoma as a guide for MSC differentiation trigger for osteogenesis and bone healing [61]. In our system, the level of mineralization appeared to be independent of oxygen tension, since we observed a similar Von Kossa and collagen type I staining for the groups differentiated under 2% and 20% O_2_. We assume that the discrepancies between our study and previous studies could be due to the oxygen concentration used during the expansion process, to the variety of MSCs species/sources used and to the choice of 3D or 2D environments in pellet or scaffold cultures. In particular, material-based differences might play a role. In our study, we investigated hMSCs, expanded under 20% O_2_ and differentiated within 3D fibrin constructs under low and high oxygen. Although it is still challenging to make broad conclusions regarding the role of oxygen on MSC biology, our study indicates that low oxygen is beneficial for chondrogenic differentiation but had little effect on osteogenic differentiation at the protein level. The lack of changes in HIF1α expression makes full interpretation of the data difficult, however the consistency of the changes suggest a HIF1α independent mechanism may be involved. The easy modulation of the oxygen tension in the in vitro studies make this a parameter that can be tailored to either stabilize a chondrogenic phenotype under low oxygen for use in cartilage repair therapies or to promote endochondral bone repair strategies under medium to high oxygen through the induction of the hypertrophy of cartilaginous grafts.

This study is not without limitations, while it is an advantage to study individual donors, the donor variation suggests studies with primary human cells may need more than the four donors used here. While that is an ideal scenario, it is demanding due to the large number of groups and cell number requirements involved. This also influenced the decision to perform one time point, as additional time points would lead to unfeasible cell numbers being required. A more detailed protein analysis of the conditioned media by way of ELISA may also provide a more detailed insight into the relative matrix retention rates. However, as a proof of concept this study does demonstrate the feasibility of such an approach. Also, the two-week culture period only allows for investigation into early events. As previous studies have shown that early events are key in the differentiation pathway [62,63], we believe that longer-term studies would strengthen the conclusions. Furthermore, a future step would be to co-culture pre-differentiated scaffolds to allow for tissue cross-talk, with the aim to enhance tissue formation. A further development of the system would be to culture the cells under osteogenic and chondrogenic conditions for two weeks, and then combine the scaffolds for a further two weeks with direct contact. This would allow for cross-talk studies to investigate if the cells at different differentiation states interact by way of soluble signals to further regulate tissue maturation. Due to the asymmetrically seeded cartilage layer, combination with an osteogenically induced base layer would lead to a tri-layered structure that loosely mimics an osteochondral plug. In case of clinical implantation, the two constructs could be attached using fibrin glue or be held in place using a subchondral bone anchor, thereby providing a pre-differentiated bilayer scaffold for osteochondral repair.

## Figures and Tables

**Table 1:** Human oligonucleotide primers and probes used for qRT-PCR.

Gene	Primer Forward (5′−3′)	Primer Reverse (5′−3′)	Probe (5′ FAM- 3′ TAMRA)
COL2A1	5′-GGC AAT AGC AGG TTC ACG TAC A-3′	5′-GAT AAC AGT CTT GCC CCA CTT ACC-3′	5′-CCT GAA GGA TGG CTG CAC GAA ACA TAC-3′
COL10A1	5′-ACG CTG AAC GAT ACC AAA TG-3′	5′-TGC TAT ACC TTT ACT CTT TAT GGT GTA-3′	5′-ACT ACC CAA CAC CAA GAC ACA GTT CTT CAT TCC-3′
ACAN	5′-AGT CCT CAA GCC TCC TGT ACT CA-3′	5′-CGG GAA GTG GCG GTA ACA-3′	5′-CCG GAA TGG AAA CGT GAA TCA GAA TCA ACT-3′
RUNX2	5′-AGC AAG GTT CAA CGA TCT GAG AT-3′	5′-TTT GTG AAG ACG GTT ATG GTC AA-3′	5′-TGA AAC TCT TGC CTC GTC CAC TCC G-3′
OC	5′-AAG AGA CCC AGG CGC TAC CT-3′	5′-AAC TCG TCA CAG TCC GGA TTG-3′	5′-ATG GCT GGG AGC CCC AGT CCC-3′
VEGF	5′-GCC CAC TGA GGA GTC CAA CA-3	5′-TCCTATGTG CTG GCC TTG GT-3′	5′-CAC CAT GCA GAT TAT GCG GAT CAA ACC T-3′
RPLP0	5′-TGG GCA AGA ACA CCA TGA TG-3′	5′-CGG ATA TGA GGC AGC AGT TTC-3′	5′-AGG GCA CCT GGA AAA CAA CCC AGC-3′

RPLP0, ribosomal protein large P0 housekeeping gene; COL2A1, collagen type 2; COL10A1, collagen type 10; ACAN, aggrecan; RUNX2, runt-related transcription factor 2; OC, osteocalcin; VEGF, Vascular Endothelial Growth Factor.

## Data Availability

Data is contained within the article. More detailed data can be provided upon reasonable request.
